# A Unifying Probabilistic View of Associative Learning

**DOI:** 10.1371/journal.pcbi.1004567

**Published:** 2015-11-04

**Authors:** Samuel J. Gershman

**Affiliations:** Department of Psychology and Center for Brain Science, Harvard University, Cambridge, Massachusetts, United States of America; University College London, UNITED KINGDOM

## Abstract

Two important ideas about associative learning have emerged in recent decades: (1) Animals are Bayesian learners, tracking their uncertainty about associations; and (2) animals acquire long-term reward predictions through reinforcement learning. Both of these ideas are normative, in the sense that they are derived from rational design principles. They are also descriptive, capturing a wide range of empirical phenomena that troubled earlier theories. This article describes a unifying framework encompassing Bayesian and reinforcement learning theories of associative learning. Each perspective captures a different aspect of associative learning, and their synthesis offers insight into phenomena that neither perspective can explain on its own.

## Author Summary

How do we learn about associations between events? The seminal Rescorla-Wagner model provided a simple yet powerful foundation for understanding associative learning. However, much subsequent research has uncovered fundamental limitations of the Rescorla-Wagner model. One response to these limitations has been to rethink associative learning from a normative statistical perspective: How would an ideal agent learn about associations? First, an agent should track its uncertainty using Bayesian principles. Second, an agent should learn about long-term (not just immediate) reward, using reinforcement learning principles. This article brings together these principles into a single framework and shows how they synergistically account for a number of complex learning phenomena.

## Introduction

Learning to predict rewards (or punishments) from the occurrence of other stimuli is fundamental to the survival of animals. When such learning occurs, it is commonly assumed that a stimulus-reward association is stored in memory [1, 2]. Two ideas have, over the last few decades, altered our understanding of how such associations are formed, and the nature of their content. First, Bayesian theories of learning have suggested that animals estimate not only the strength of associations, but also their uncertainty in these estimates [3–8]. Second, reinforcement learning (RL) theories have suggested that animals estimate long-term cumulative future reward [9–11].

Both Bayesian and RL theories can be viewed as generalizations of the seminal Rescorla-Wagner model [12] that address some of its limitations. The mathematical derivations of these generalizations and their empirical support will be reviewed in the following sections. Bayesian and RL theories are derived from different—but not mutually exclusive—assumptions about the nature of the learning task. The goal of this paper is to unify these perspectives and explore the implications of this unification.

One set of assumptions about the learning task concerns the target of learning. The Bayesian generalization of the Rescorla-Wagner model, embodied in the Kalman filter [3, 4, 6], assumes that this is the problem of predicting immediate reward, whereas RL theories, such as temporal difference (TD) learning, assume that the goal of learning is to predict the cumulative future reward. A second set of assumptions concerns the representation of uncertainty. The Kalman filter learns a Bayesian estimator (the posterior distribution) of expected immediate reward, whereas TD learns a point estimator (a single value rather than a distribution) of expected future reward. As shown below, the Rescorla-Wagner model can be construed as a point estimator of expected immediate reward.

**Fig 1:**
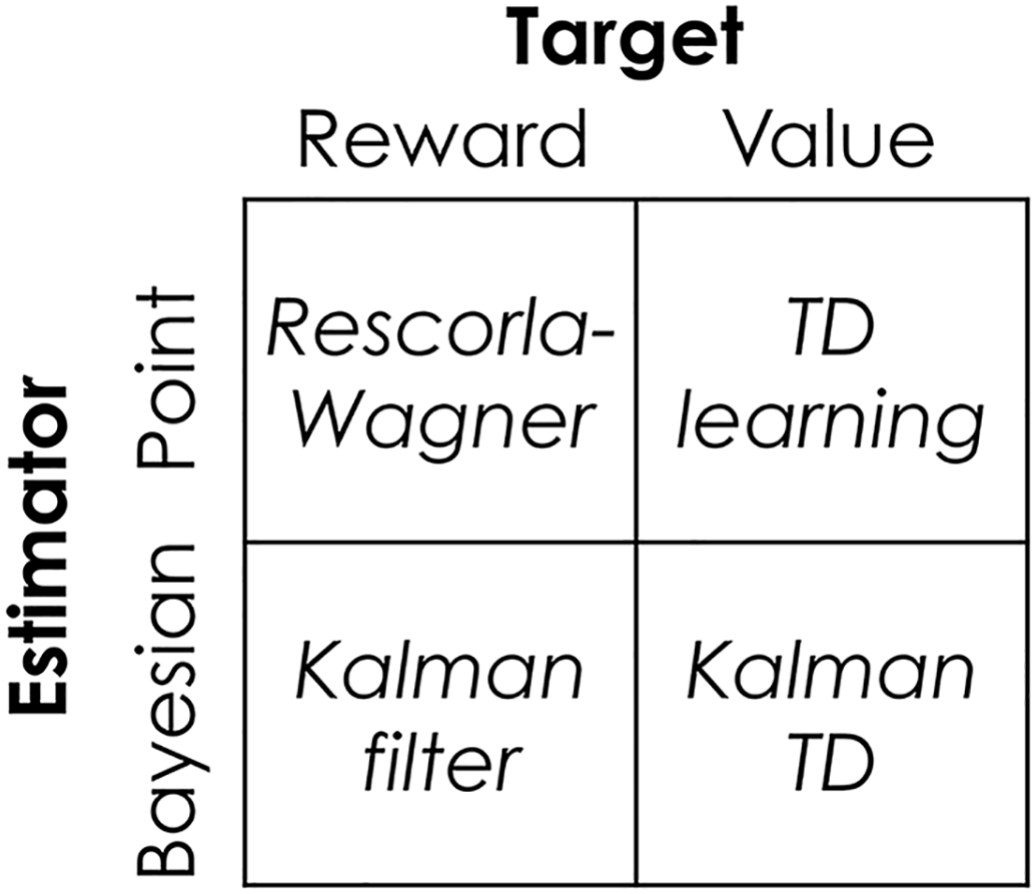
Organizing Bayesian and reinforcement learning theories. Point estimation algorithms learn the expected reward or value, while Bayesian algorithms learn a posterior distribution over reward or value. The columns show *what* is learned, and the rows show *how* it is learned.

After reviewing these different modeling assumptions (organized in Fig 1), I show how they can be naturally brought together in the form of the Kalman TD model. This model has been previously studied in the RL literature [13], but has received relatively little attention in neuroscience or psychology (see [14] for an exception). I explain how this model combines the strengths of Bayesian and TD models. I will demonstrate this point using several experimental examples that neither model can account for on its own.

## Results

### Preliminaries

Let **x**_*n*_ denote the vector of conditioned stimulus (CS) intensities on trial *n* (all vectors are taken to be column vectors), **w**_*n*_ denote the associative strengths (or weights), and *r*_*n*_ denote the unconditioned stimulus (US; i.e., observed reward). Note that traditional associative learning theories interpret *r*_*n*_ as the asymptotic level of responding supported by the US on the current trial; however, in this article I interpret *r*_*n*_ as reward in order to facilitate the connection to RL.

To compactly describe experimental paradigms, I use uppercase letters (A, B, etc.) to denote conditioned stimuli, and combinations of letters (e.g., AB) to denote stimulus compounds. A stimulus (or compound) terminating in reward is denoted by A→+. Similarly, a stimulus terminating in no reward is denoted by A→-. A stimulus terminating with the onset of another stimulus is denoted A→B. The notation A→? indicates that conditioned responding to A is the dependent measure in a particular experiment. When multiple trial types are interleaved within a phase, forward slashes are used (e.g., A→+ / B→-), and contiguous phases are separated by semi-colons (e.g., A→+; B→-).

Making predictions about empirical phenomena is complicated by the fact that experimental paradigms use diverse stimuli, rewards, and behavioral measures. The simulations reported below are predicated on the assumption that we can abstract away from some of these experimental details and predict response rates simply on the basis of reward expectation, as acquired by trial-and-error learning. This assumption is certainly false: response rates depend on other factors, such as motivation and stimulus-specific properties (e.g., [15]). Nonetheless, this assumption enables the models considered below to make predictions about a wide range of experimental paradigms without getting bogged down in experimental minutiae. The same is true for many other computational models, and is helpful for making progress before more realistic theoretical assumptions can be refined.

### The Rescorla-Wagner model

The Rescorla-Wagner model is the cornerstone of modern associative learning theory. While it has a number of crucial shortcomings [16], the model stimulated decades of experimental research and served as the basis of more sophisticated models [17–19]. Learning is governed by the following equation:

$$w_{n+1}=w_{n}+\alpha x_{n}\delta_{n},$$ (1)

$$v_{n}=w_{n}^{⊤}x_{n}$$ (2)

where *α* ∈ [0, 1] is a learning rate parameter (also known as *associability*), *δ*_*n*_ = *r*_*n*_ − *v*_*n*_ is the *prediction error*, and *v*_*n*_ is the reward expectation, which is taken to be monotonically related to the conditioned response.

In the next section, I describe a probabilistic interpretation of this learning rule, which will play an important role in subsequent developments. I then discuss some empirical implications of the model.

#### Probabilistic interpretation

To derive a probabilistic interpretation, we need to impute to the animal a set of probabilistic assumptions about how its sensory data are generated—the animal’s internal model. Specifically, the internal model is defined by a *prior* on weights, *p*(**w**_0_), a *change process* on the weights, *p*(**w**_*n*_∣**w**_*n*−1_), and a reward distribution given stimuli and weights, *p*(*r*_*n*_∣**w**_*n*_, **x**_*n*_). Following earlier work [3, 4, 6], I take this to be a linear-Gaussian dynamical system (LDS):

$$w_{0}∼N\left(0,\sigma_{w}^{2}I\right)$$ (3)

$$w_{n}∼N\left(w_{n-1},\tau^{2}I\right)$$ (4)

$$r_{n}∼N\left(v_{n},\sigma_{r}^{2}\right),$$ (5)

where **I** is the identity matrix. Intuitively, the LDS makes the following claims about the animal’s internal model. First, the prior on weights posits that weights tend to be close to 0 (i.e., associations tend to be weak); the strength of this prior is inversely proportional to $\sigma_{w}^{2}$. Second, the change process posits that weights tend to change slowly and independently over time; the volatility of this change process increases with *τ* ^2^. Third, the reward distribution posits that reward is a noisy linear combination of stimulus activations.

From the animal’s perspective, the goal of learning is to recover an estimate of the weights. The generative process serves as a set of soft constraints on the weight estimator. In other words, the generative process provides an inductive bias that makes some estimators better than others. In order to precisely define what makes an estimator “better,” we need to specify an objective function that is maximized by the optimal estimator. Let us first make the simplifying assumption that the weights do not change over time (i.e., *τ* ^2^ = 0), in which case the weights are static parameters and we can drop the trial index. Under this assumption, it can be shown that the objective function maximized (asymptotically as *t* → ∞) by the Rescorla-Wagner model is the log-likelihood log *p*(*r*_1:*n*_∣**w**, **x**_1:*t*_), where the index 1:*n* denotes all trials from 1 to *n*.

To show this, I draw a connection between the Rescorla-Wagner model and the Robbins-Monro algorithm for stochastic approximation [20]. In the context of the LDS described above, the Robbins-Monro algorithm updates the weight estimate $\hat{w}$ according to:

$${\hat{w}}_{n+1}={\hat{w}}_{n}+\alpha_{n}\sigma^{-2}x_{n}\left(r_{n}-v_{n}\right),$$ (6)

where *α*_*n*_ is a dynamically decreasing learning rate satisfying

$$\sum_{n=0}^{\infty }\alpha_{n}=\infty ,\;\;\sum_{n=0}^{\infty }\alpha_{n}^{2}<\infty .$$ (7)

One simple choice of learning rate that satisfies these conditions is *α*_*n*_ = 1/*n*. The Robbins-Monro algorithm converges asymptotically to the maximum likelihood estimate of **w**. Comparing Eqs 1 and 6 (and allowing *σ* ^−2^ to be absorbed into the learning rate), it can be seen that the Rescorla-Wagner model with a dynamically decreasing learning rate is a maximum likelihood estimator (see also [21]). This analysis echoes the observation that the Rescorla-Wagner model is an instantiation of the “least mean squares” (aka Widrow-Hoff) learning rule [22]: under a Gaussian observation model, minimizing summed squared error is equivalent to maximizing likelihood. The main difference is that the least mean squares rule assumes a static learning rate, and imposes restrictions on the learning rate to ensure convergence.

While the Rescorla-Wagner model thus has a normative basis in statistical estimation, it is not a fully probabilistic estimator—it only maintains a single “point” hypothesis about the weights. As a consequence, the estimator ignores uncertainty about the weights. There is good evidence that the brain maintains representations of uncertainty [23], and updates these representations using Bayesian inference [24]. Below I discuss a Bayesian generalization of the Rescorla-Wagner model, following a brief consideration of the empirical phenomena that motivate this generalization.

#### Empirical implications

The Rescorla-Wagner model formalizes two important principles: (1) learning is driven by reward prediction errors; and (2) simultaneously presented stimuli summate to predict reward. These principles will figure prominently in the subsequent discussion of the model’s limitations and possible remedies.

To see that learning is driven solely by reward prediction errors, notice that **w**_*n*_ is updated only when the prediction error is non-zero. One surprising consequence of this property is that associative strength can in some cases *weaken* as a consequence of reinforcement. For example, Rescorla [25] demonstrated that reinforcing a compound consisting of two previously reinforced stimuli caused a decrement in responding to the individual stimuli on a subsequent test. This effect is referred to as *overexpectation* because summing the associative strength of two individually reinforced stimuli should produce a larger reward prediction than either stimulus alone. Because the reinforcer magnitude is the same, the prediction error will be negative, and thus the associative strength for both stimuli will be decremented. This demonstrates that learning is driven not by reinforcement *per se*, but by *unexpected* reinforcement.

The same principles can give rise to negative (inhibitory) associative strength. In the *conditioned inhibition* paradigm [26, 27], A→+ trials are interspersed with AB→- trials, resulting in negative associative strength accruing to stimulus B (as assessed, for example, by showing that pairing B with a previously reinforced stimulus C reduces responding relative to C alone). According to the Rescorla-Wagner model, the negative association is acquired because of the negative prediction error on AB→- trials; B must have a negative weight in order to counterbalance the excitatory weight of A.

The combination of error-driven learning with associative summation leads to stimulus competition. For example, in *forward (Kamin) blocking*[28], stimulus A is paired with reward and then in a second phase the compound AB is paired with reward. In a subsequent test of B alone, responding is lower compared to a condition in which the first phase is omitted. In terms of the Rescorla-Wagner model, stimulus A *blocks* acquisition of an association between B and reward because the reward is fully predicted by A and hence there is no prediction error to drive learning in the second phase. A similar argument accounts for the phenomenon of *overshadowing*[26], in which reinforcing the compound AB results in weaker responding to the individual stimulus elements compared to a condition in which each stimulus is reinforced separately.

Although considerable evidence supports the existence of error-driven learning and stimulus competition in associative learning, violations of these principles are well-documented [16]. For example, presenting a stimulus alone prior to pairing it with reward retards acquisition of the stimulus-reward association, a phenomena known as the *CS pre-exposure effect* or *latent inhibition*[29]. Because the associative strength is presumably initialized to 0, the prediction error is 0 during pre-exposure and hence no associative learning should occur according to the Rescorla-Wagner model. Another example of learning in the absence of prediction errors is second-order conditioning [26, 30]: The serial compound A→B results in conditioning of A if B was previously paired with reward. Here again there is no prediction error during the A→B and hence no learning should have occurred (a more fundamental problem here, which I discuss further below, is that the Rescorla-Wagner model only makes trial-level predictions and hence is actually inapplicable to serial-compound conditioning).

The Rescorla-Wagner model also runs into trouble in situations where absent stimuli appear to compete with present stimuli. For example, in backward blocking [31–33], a compound AB is reinforced and then A is reinforced by itself, resulting in a reduction of responding to B alone. Conversely, stimulus competition can be reduced by post-training extinction of one element [34–36].

These findings undercut some of the basic claims of the Rescorla-Wagner model, and have stimulated extensive work in animal learning theory [2]. The next two sections will focus on two normatively-motivated generalizations of the Rescorla-Wagner model that can accommodate these (and many other) findings, before proceeding to a unifying view of these generalizations.

### Bayesian inference and the Kalman filter

The probabilistic interpretation of the Rescorla-Wagner model given above shows that it is a maximum likelihood estimator of the weight vector. This estimator neglects the learner’s uncertainty by only representing the single most likely weight vector. Given that humans and other animals are able to report their uncertainty, and that these reports are often well-calibrated with veridical confidence (i.e., the probability of being correct; see [37]), it appears necessary to consider models that explicitly represent uncertainty. Moreover, such models are an important step towards understanding how the brain represents uncertainty [23, 24].

Bayesian models of learning posit that the learner represents uncertainty in the form of a posterior distribution over hypotheses given data. In the case of associative learning, the posterior distribution is stipulated by Bayes’ rule as follows:

$$p\left(w_{n}|x_{1:n}\right)\propto p\left(x_{1:n}|w_{n}\right)p\left(w_{n}\right).$$ (8)

Under the LDS specified in Eqs 3–5, the posterior is Gaussian with mean ${\hat{w}}_{n}$ and covariance matrix Σ_*n*_, updated using the Kalman filter equations:

$${\hat{w}}_{n+1}={\hat{w}}_{n}+k_{n}\delta_{n}$$ (9)

$$\Sigma_{n+1}=\Sigma_{n}+\tau^{2}I-k_{n}x_{n}^{⊤}\left(\Sigma_{n}+\tau^{2}I\right),$$ (10)

where ${\hat{w}}_{0}=0$, $\Sigma_{0}=\sigma_{w}^{2}I$, and **k**_*n*_ is the *Kalman gain*:

$$k_{n}=\frac{\left(\Sigma_{n}+\tau^{2}I\right)x_{n}}{x_{n}^{⊤}\left(\Sigma_{n}+\tau^{2}I\right)x_{n}+\sigma_{r}^{2}}.$$ (11)

Here the Kalman gain has replaced the learning rate *α* in the Rescorla-Wagner model. Importantly, the Kalman gain is stimulus-specific, dynamic and grows monotonically with the uncertainty encoded in the diagonals of the posterior covariance matrix Σ_*n*_. This allows the Kalman filter model to explain some of the phenomena that are problematic for the Rescorla-Wagner model.

Two factors govern the covariance matrix update. First, uncertainty grows over time due to the random diffusion of the weights (Eq 4); this is expressed by the *τ* ^2^ **I** term in Eq 10. The growth of uncertainty over time increases with the diffusion variance *τ* ^2^, leading to higher learning rates in more “volatile” environments. The relationship between volatility and learning rate follows intuitively from the fact that high volatility means that older information is less relevant and can therefore be forgotten [38, 39]. The second factor governing the covariance matrix update is the reduction of uncertainty due to observation of data, as expressed by the term $k_{n}x_{n}^{⊤}\left(\Sigma_{n}+\tau^{2}I\right)$. Whenever a cue is observed, its variance on the diagonal of the covariance matrix is reduced, as are the covariances (off-diagonals) for any correlated cues.

**Fig 2:**
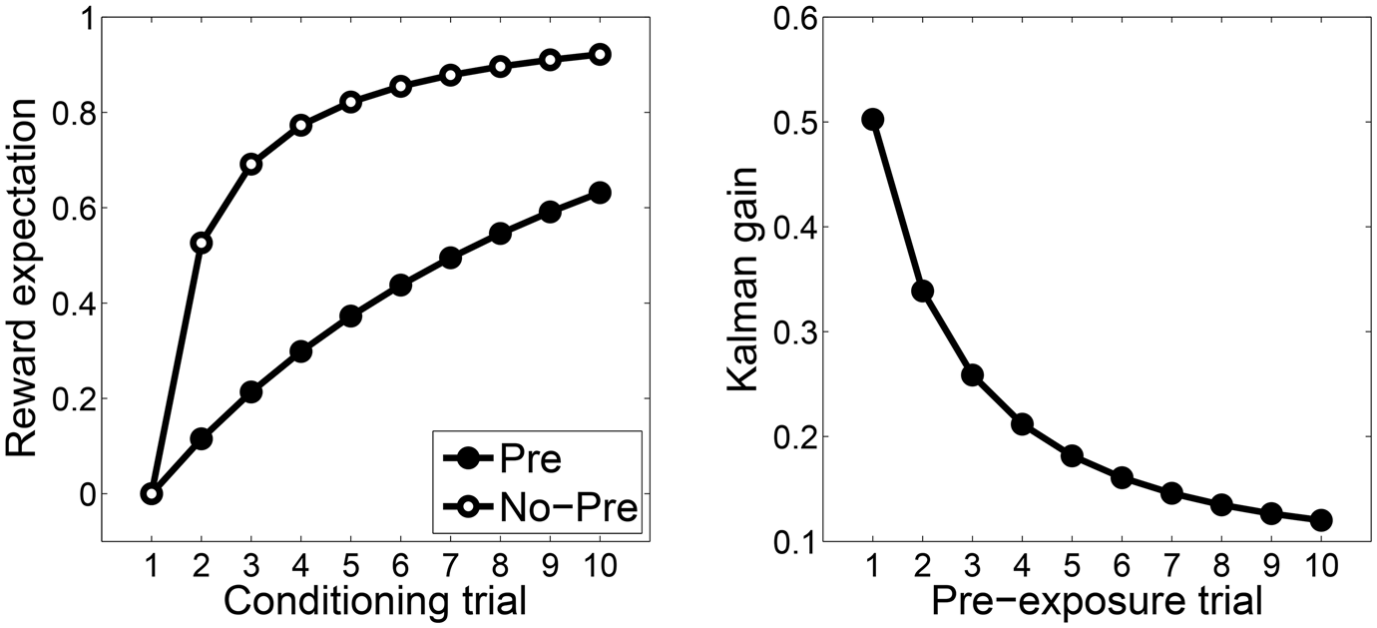
Kalman filter simulation of latent inhibition. (*A*) Reward expectation following pre-exposure (Pre) and no pre-exposure (No-Pre) conditions. (*B*) The Kalman gain as a function of pre-exposure trial.

One implication of the Kalman filter is that repeated CS presentations will attenuate posterior uncertainty and therefore reduce the Kalman gain. As illustrated in Fig 2, this reduction in gain produces latent inhibition, capturing the intuition that CS pre-exposure reduces “attention” (associability or learning rate). The Kalman filter can also explain why interposing an interval between pre-exposure and conditioning attenuates latent inhibition [40]: The posterior variance grows over the interval (due to random diffusion of the weights), increasing the Kalman gain. Thus, the Kalman filter can model some changes in learning that occur in the absence of prediction error, unlike the Rescorla-Wagner model.

The Kalman filter can also account for the effects of various post-training manipulations, such as backward blocking [3, 6]. During the compound training phase, the model learns that the cue weights must sum to 1 (the reward value), and thus any weight configurations in which one weight is large necessitates that the other weight be small. Mathematically, this is encoded as negative covariance between the weights (i.e., the off-diagonals of Σ_*n*_). As a consequence, learning that *A* predicts reward leads to a reduction in the associative strength for *B*.

**Fig 3:**
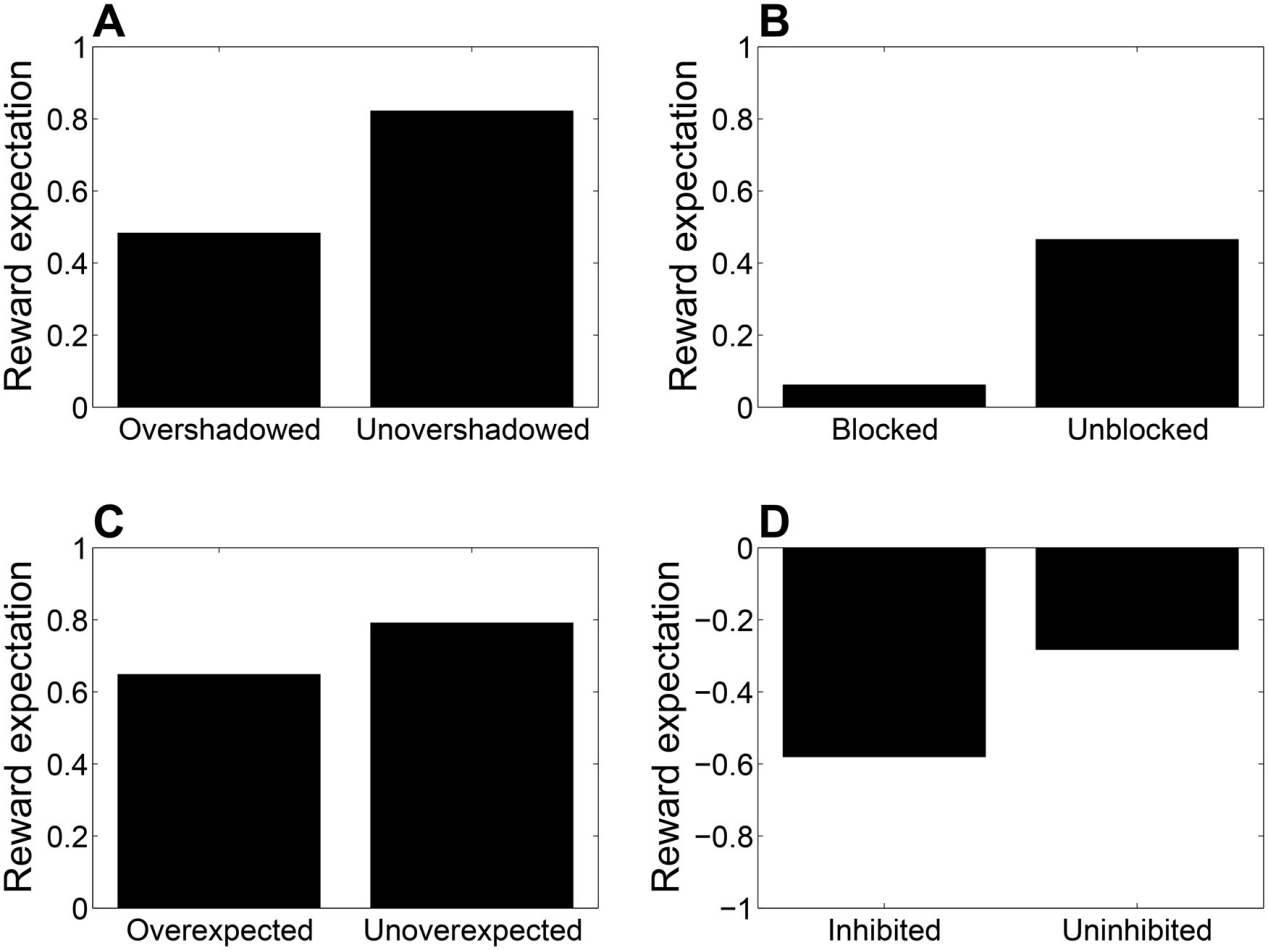
Kalman filter simulation of recovery phenomena. (*A*) Overshadowing and unovershadowing by extinction of the overshadowing stimulus. (*B*) Forward blocking and unblocking by extinction of the blocking stimulus. (*C*) Overexpectation and unoverexpectation by extinction of one element. (*D*) Conditioned inhibition and uninhibition by extinction of the excitatory stimulus.

Beyond backward blocking, the Kalman filter can capture a wider range of recovery phenomena than has previously been simulated. Four examples are shown in Fig 3 (see Methods for simulation details). As shown by Matzel and colleagues [34], overshadowing (AB→+ training leads to weaker responding to B compared to B→+ training) can be counteracted by extinguishing one of the stimulus elements prior to test (AB→+; A→-). Similarly, extinguishing the blocking stimulus in a forward blocking paradigm (A→+; AB→+; A→-; B→?) causes a recovery of responding to the blocked stimulus [35], and extinguishing one of the stimulus A in an overexpectation paradigm (A→+ / B→+; AB→+; A→-; B→?) causes a recovery of responding to the other stimulus B [36]. Finally, extinguishing the excitatory stimulus A in a conditioned inhibition paradigm (A→+ / AB→-; A→-) reduces the negative associative strength of the inhibitory stimulus B [41].

All of these examples have a common structure shared with backward blocking, where compound training causes the acquisition of negative covariance between the stimulus elements. This negative covariance implies that post-training inflation or deflation of one stimulus will cause changes in beliefs about the other stimulus. Post-training recovery phenomena have inspired new theories that allow learning to occur for absent stimuli. For example, Van Hamme and Wasserman [18] developed an extension of the Rescorla-Wagner model in which the associative strengths for absent cues are modified just like present cues, but possibly with a smaller learning rate (see also [19, 42, 43]). The Kalman filter provides a normative explanation of recovery phenomena, while retaining close similarities with classical theories like the Rescorla-Wagner model.

### Temporal difference learning and long-term reward prediction

The Kalman filter fixes some of the problems vexing the Rescorla-Wagner model, but a fundamental limitation remains: The Rescorla-Wagner model is a *trial-level* model, which means that it only makes predictions at the granularity of a trial, remaining blind to intra-trial structure such as stimulus duration and the inter-stimulus interval. While one can finesse this by treating each time-step in the model as a sub-division of a trial, such a solution is inadequate because it fails to capture the fact that conditioned responses are anticipatory of long-term future events. For example, interposing a delay between CS offset and US onset means that the CS never co-occurs with the US and hence should not produce any conditioning according to this particular real-time extension of the Rescorla-Wagner model (contrary to the empirical data).

It is possible to augment the Rescorla-Wagner model with a time-varying stimulus trace evoked by the CS, allowing the trace to enter into association with the US. This idea goes back to the work of Pavlov [26] and Hull [44], who posited that the stimulus trace persists for several seconds following CS offset, decaying gradually over time. More complex stimulus traces have been explored by later researchers (e.g., [45, 46]).

While a persistent trace enables the model to capture aspects of intra-trial temporal structure, there is an additional problem: the association between the trace and the US can only be reinforced following US presentation, but contrary to this assumption it has been demonstrated empirically that an association can be reinforced without any pairing between the CS and US. As mentioned above, an example is second-order conditioning [26, 30], where A is paired with reward and subsequently B is paired with A, resulting in conditioned responding to B. An analogous phenomenon, known as *conditioned reinforcement*, has been studied in operant conditioning [47]. Somehow, a CS must be able to acquire the reinforcing properties of the US with which it has been paired.

The TD model [9] offers a solution to both of these problems, grounded in a different rational analysis of associative learning. The underlying assumption of the TD model is that the associative learning system is designed to learn a prediction of *long-term future* reward, rather than *immediate* reward (as was assumed in our rational analysis of the Rescorla-Wagner and Kalman filter models). Specifically, let us imagine an animal that traverses a “state space” defined by the configuration of stimuli, moving from **x**_*t*_ at time *t* to **x**_*t*+1_ according to a transition distribution *p*(**x**_*t*+1_∣**x**_*t*_). (Note that we now index by *t* to emphasize that we are in “real time”). The *value* of state **x**_*t*_ is defined as the expected discounted future return (cumulative reward):

$$V\left(x_{t}\right)=E\;[\sum_{k=0}^{\infty }\gamma^{k}r_{t+k}],$$ (12)

where *γ* ∈ [0, 1] is a *discount factor* that controls how heavily the near future is weighted relative to the distant future. Applications of the TD model to associative learning assume that conditioned responding is monotonically related to the animal’s value estimate. This means that two stimuli might have the same expected reward, but responding will be higher to the stimulus that predicts greater cumulative reward in the future.

The RL problem is to learn the value function. As is common in the RL literature [48, 49], I will assume that the value function can be approximated as a linear combination of stimuli: $V\left(x_{t}\right)=w_{t}^{⊤}x_{t}$. This reduces the RL problem to learning **w**_*t*_. This can be accomplished using an update very similar to that of the Rescorla-Wagner model [49]:

$${\hat{w}}_{t+1}={\hat{w}}_{t}+\alpha x_{t}\delta_{t},$$ (13)

where *δ*_*t*_ is now defined as the *temporal difference prediction error*:

$$\delta_{t}=r_{t}+\gamma {\hat{w}}_{t}^{⊤}x_{t+1}-{\hat{w}}_{t}^{⊤}x_{t}.$$ (14)

Except for the addition of the future reward expectation term $\gamma {\hat{w}}_{t}^{⊤}x_{t+1}$, the TD prediction error is identical to the Rescorla-Wagner prediction error, and reduces to it when *γ* = 0.

In order to apply the TD model to associative learning tasks, it is necessary to specify a temporally extended stimulus representation. Sutton and Barto [9] adopted the *complete serial compound* (CSC) representation, which divides a stimulus into a sequence of non-overlapping bins. Thus, a stimulus lasting for two time steps would be represented by **x**_1_ = [1, 0] and **x**_2_ = [0, 1]. Although there are a number of problems with this representation [11, 50–52], I use it here for continuity with previous work.

The TD model can account for a number of intra-trial phenomena, such as the effect of stimulus timing on acquisition and cue competition (see [9, 11] for extensive simulations). It also provides a natural explanation for second-order conditioning: despite the immediate reward term *r*_*t*_ in Eq 14 being 0 for A→B trials, the future reward expectation term $\gamma {\hat{w}}_{t}^{⊤}x_{t+1}$ is positive (due to the B→+ trials) and hence the value of A is increased.

In summary, the TD model has proven to be a successful real-time generalization of the Rescorla-Wagner model, and also has the advantage of being grounded in the normative theory of RL. However, it lacks the uncertainty-tracking mechanisms of the Kalman filter, which I argued are important for understanding CS pre-exposure and post-training recovery effects. I now turn to the problem of unifying the Kalman filter and TD models.

### A unifying view: Kalman temporal difference learning

Bayesian versions of TD learning have been developed in a number of different forms [13, 53, 54]; all of them have in common the idea that an agent tracks the entire distribution over discounted future returns, not just the mean. Of particular interest is *Kalman TD*, an elegant adaptation of the Kalman filtering machinery to TD learning developed by Geist and Pietquin [13]. Operationally, the only change from the Kalman filter model described above is to replace the stimulus features **x**_*n*_ with their discounted time derivative, **h**_*t*_ = *γ* **x**_*t* + 1_−**x**_*t*_. To see why this makes sense, note that the immediate reward can be expressed in terms of the difference between two values:

$$\begin{array}{ll} r_{t} & =\gamma V\left(x_{t+1}\right)-V\left(x_{t}\right)\\ & =\gamma w_{t}^{⊤}x_{t+1}-w_{t}^{⊤}x_{t}\\ & =w_{t}^{⊤}\left(\gamma x_{t+1}-x_{t}\right). \end{array}$$ (15)

I have assumed here, as in the previous section, that values are linear in the stimulus features. As the derivation shows, this implies that rewards are linear in the discounted time derivative of the stimulus features. Under the assumption that the weights evolve over time as a Gaussian random walk and the rewards are corrupted by Gaussian noise, we can use the same LDS formulation described earlier, for which the Kalman filter implements Bayesian estimation.

Kalman TD combines the strengths of Kalman filtering and TD learning: it is a real-time model that that represents a distribution over weights rather than a point estimate. These properties allow the model to capture both within-trial structure and retrospective revaluation. In the remainder of this section, I present several examples that illustrate the intersection of these phenomena, and compare the predictions of TD and Kalman TD (since these examples involve within-trial structure, I do not consider the Kalman filter or Rescorla-Wagner).

**Fig 4:**
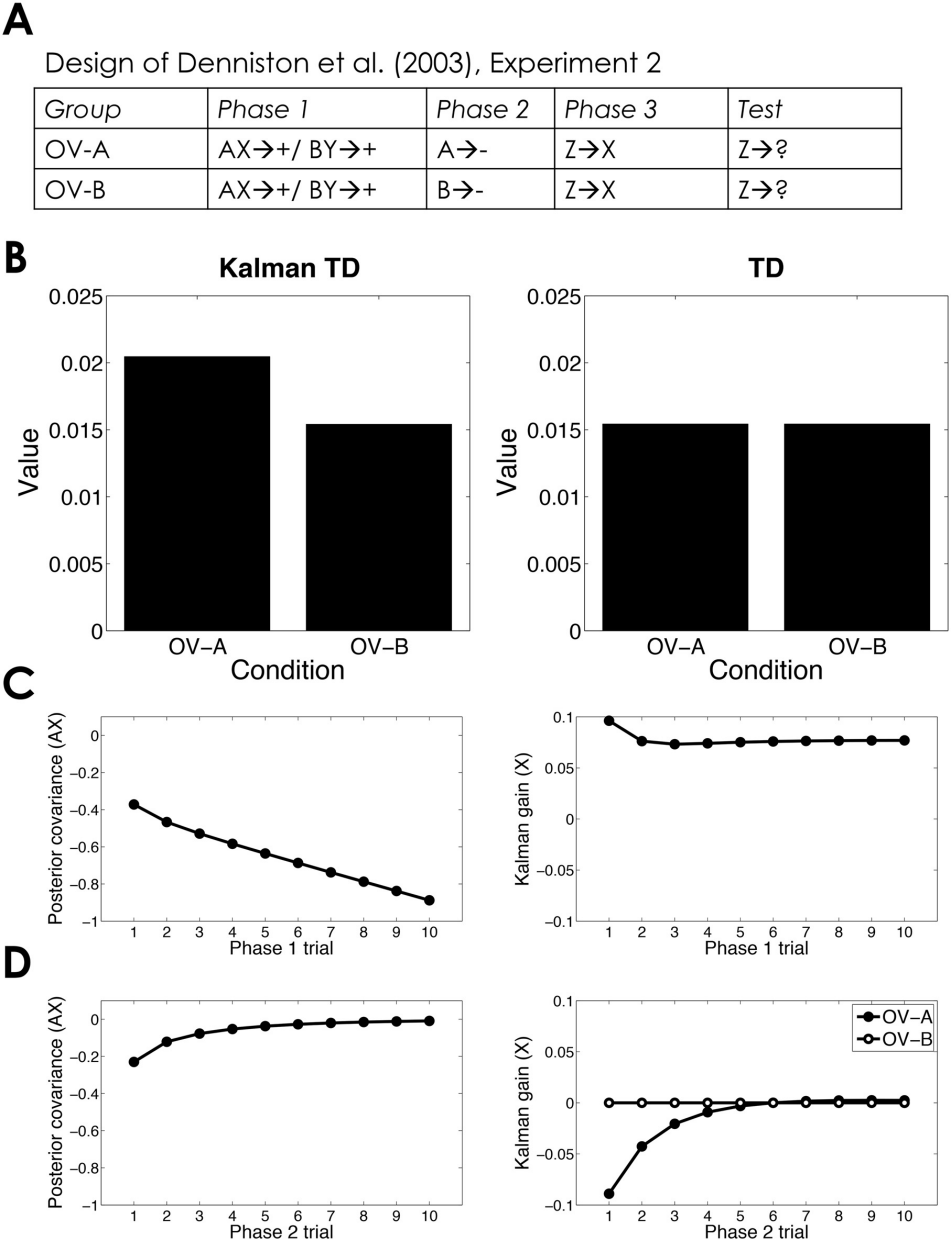
Overshadowing and second-order conditioning. (*A*) Experimental design [55]. Note that two control groups have been ignored here for simplicity. (*B*) Simulated value of stimulus Z computed by Kalman TD (left) and TD (right). Only Kalman TD correctly predicts that extinguishing an overshadowing stimulus will allow the overshadowed stimulus to support second-order conditioning. (*C*) Posterior covariance between weights for stimuli A and X (left) and Kalman gain for stimulus X (right) as a function of Phase 1 trial. (*D*) Posterior covariance between weights for stimuli A and X (left) and Kalman gain for stimulus X (right) as a function of Phase 2 trial.

Denniston et al. [55] presented a series of experiments exploring recovery from overshadowing. In one experiment (summarized in Fig 4A), the authors combined overshadowing and second-order conditioning to show that extinguishing an overshadowed stimulus allows its partner to better support second-order conditioning. Animals were divided into two groups, OV-A and OV-B. Both groups first learned to associate two light-tone compounds (AX and BY) with a US (a footshock in this case). This compound training protocol was expected to result in overshadowing. One element of the compound was then extinguished (A in group OV-A, B in group OV-B). Stimulus X was then used as a second-order reinforcer for conditioning of a novel stimulus, Z. Denniston et al. found that overshadowing reduced the ability of an overshadowed stimulus to support second-order conditioning, but this reduction could be attenuated if the overshadowing stimulus was extinguished. In particular, they found that responding at test to stimulus Z was greater in group OV-A than in group OV-B.

Simulations show that KTD, but not TD, can capture this finding (Fig 4B). While TD can capture second-order conditioning, it cannot explain why post-training extinction changes the value of an absent stimulus, because only the weights for presented stimuli are eligible for updating. The latter phenomenon is captured by the Kalman filter, which encodes the negative covariation between stimuli. As a consequence, the Kalman gain for stimulus X during Phase 2 (despite X not appearing during this phase) is negative, meaning that extinguishing A will cause inflation of X. By contrast, extinguishing B has no effect on the value of X, since B and X did not covary during Phase 1. This is essentially the same logic that explains the post-training recovery phenomena described above, but applied to a second-order conditioning scenario outside the scope of the Kalman filter.

**Fig 5:**
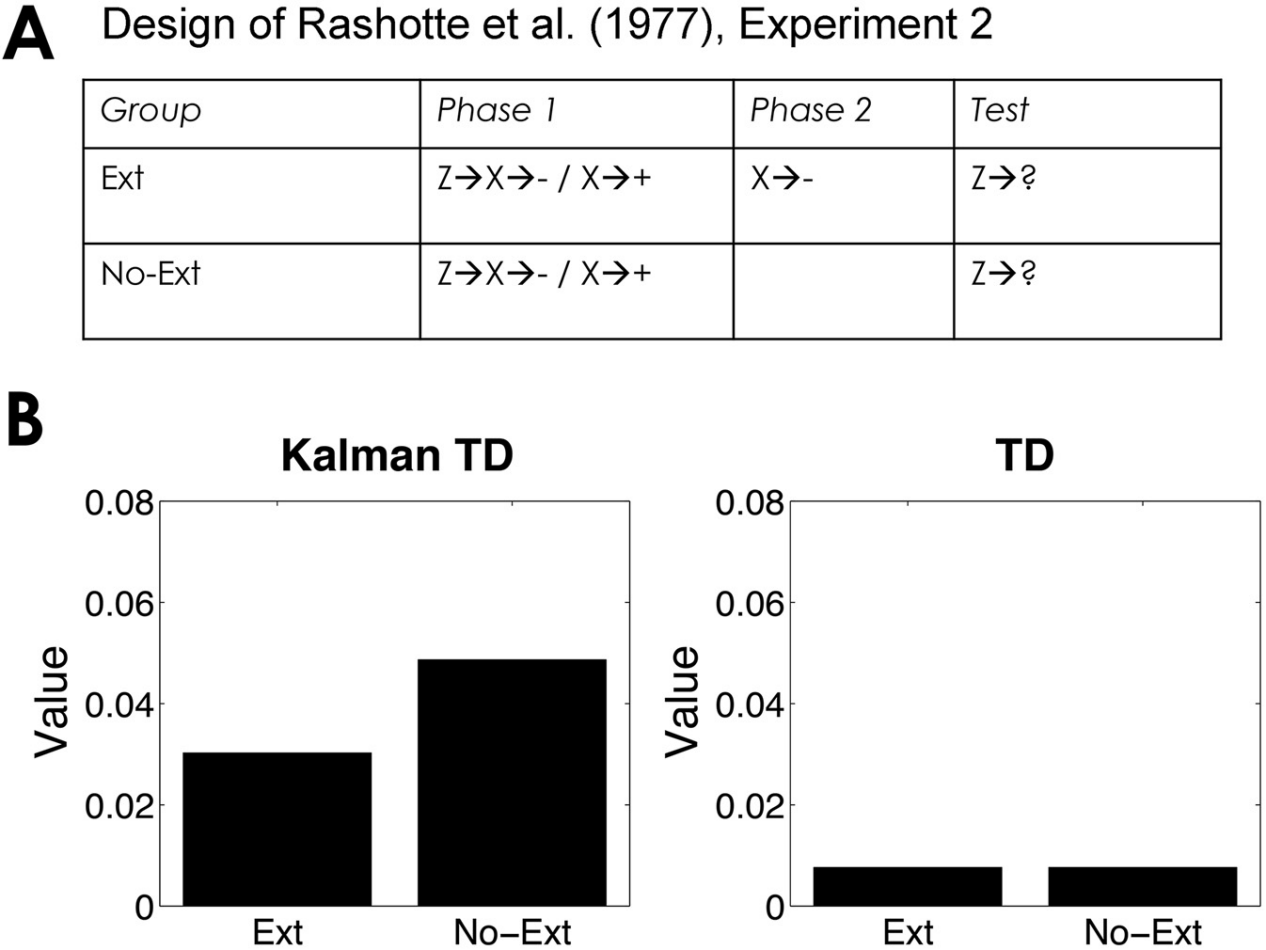
Second-order extinction. (*A*) Experimental design [56]. (*B*) Simulated value of stimulus Z computed by Kalman TD (left) and TD (right).

One extensively studied aspect of second-order conditioning has been the effect of extinguishing the first-order stimulus on responding to the second-order stimulus. Rashotte and colleagues [56] reported a Pavlovian autoshaping experiment with pigeons in which extinction of the first-order stimulus reduces responding to the second-order stimulus. This finding has been replicated a number of times [57–59], although notably it is not found in a number of other paradigms [30, 60], and a comprehensive explanation for this discrepancy is still lacking. Fig 5 shows that Kalman TD predicts sensitivity to first-order extinction, whereas TD predicts no sensitivity. The sensitivity of Kalman TD derives from the positive covariance between the first- and second-order stimuli, such that changes in the value of the first-order stimulus immediately affect the value of the second-order stimulus.

**Fig 6:**
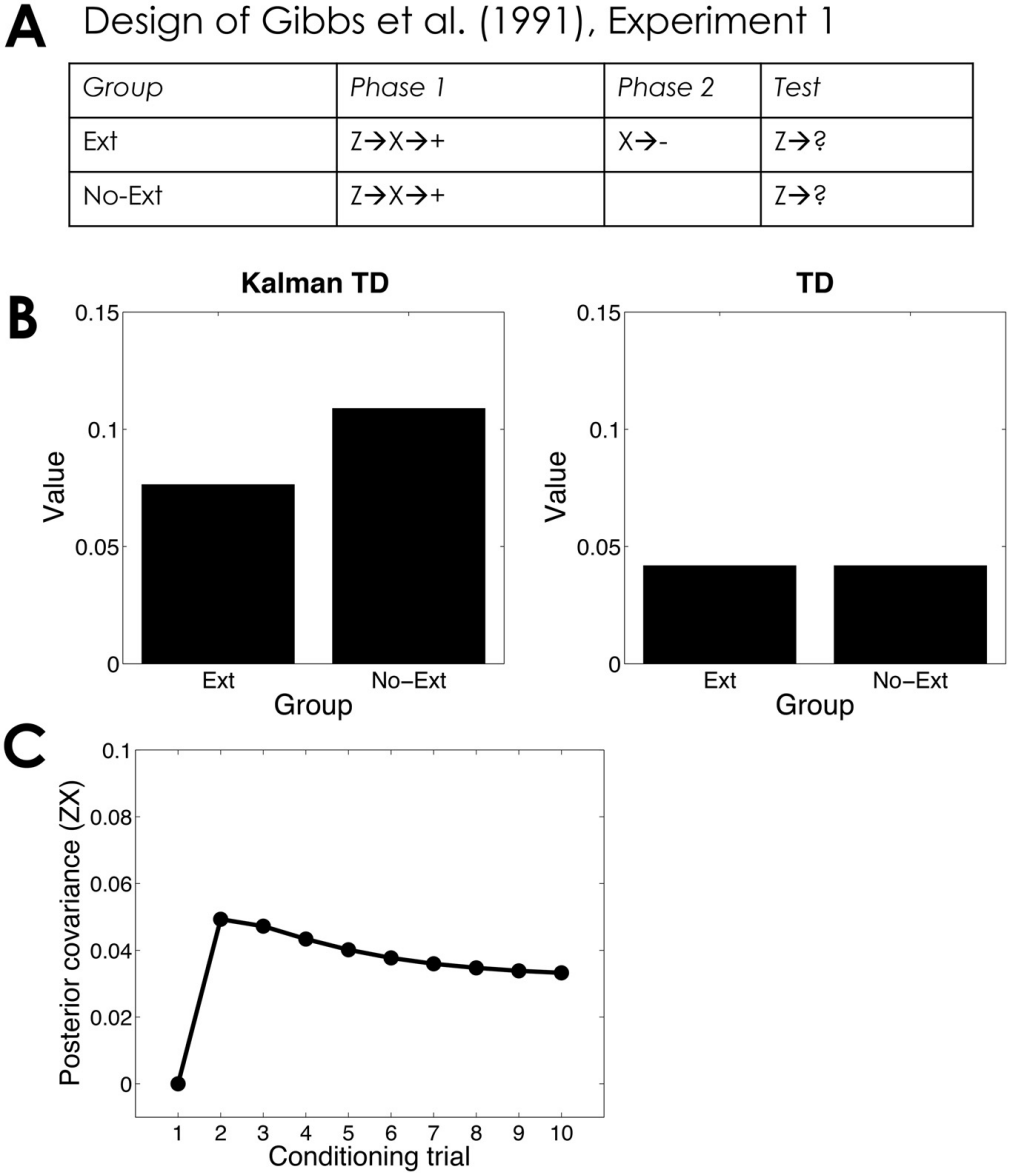
Serial compound extinction. (*A*) Experimental design [61]. (*B*) Simulated value of stimulus Z computed by Kalman TD (left) and TD (right). (*C*) Posterior covariance between the weights for stimuli Z and X as a function of conditioning trial.

I next turn to serial compound conditioning, which illustrates the within-trial behavior of Kalman TD. As summarized in Fig 6A, Gibbs et al. [61] studied the effects of extinguishing stimulus X following serial compound training (Z→X→+). They found that this extinction treatment reduced the conditioned response to Z (see [15] for similar results). Kalman TD can account for this finding (Fig 6B) because the positive covariance between Z and X means that the value of Z is sensitive to post-training manipulations of X’s value (Fig 6C). TD, which lacks a covariance-tracking mechanism, cannot account for this finding.

**Fig 7:**
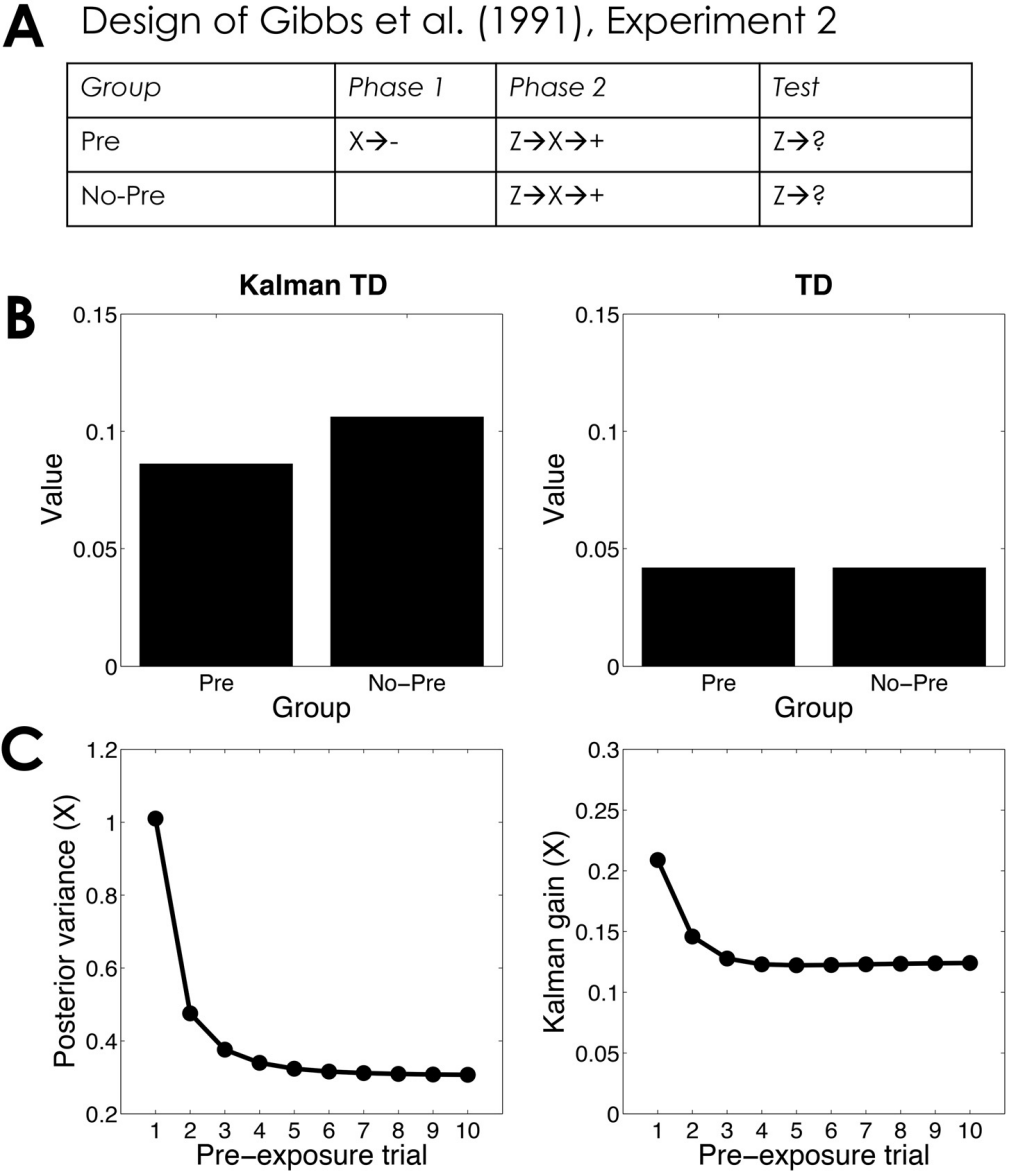
Serial compound latent inhibition. (*A*) Experimental design [61]. (*B*) Simulated value of stimulus Z computed by Kalman TD (left) and TD (right). (*C*) Posterior variance (left) and Kalman gain (right) of stimulus X as a function of pre-exposure trial.

In a second experiment (Fig 7A), Gibbs et al. had the extinction phase occur prior to training, thereby making it a latent inhibition (CS pre-exposure) design. As with the extinction treatment, latent inhibition reduces responding to Z, a finding that can be accounted for by Kalman TD, but not TD (Fig 7B). The Kalman TD account is essentially the same as the Kalman filter account of latent inhibition: Pre-exposure of X causes its posterior variance to decrease, which results in a concomitant reduction of the Kalman gain (Fig 7C).

**Fig 8:**
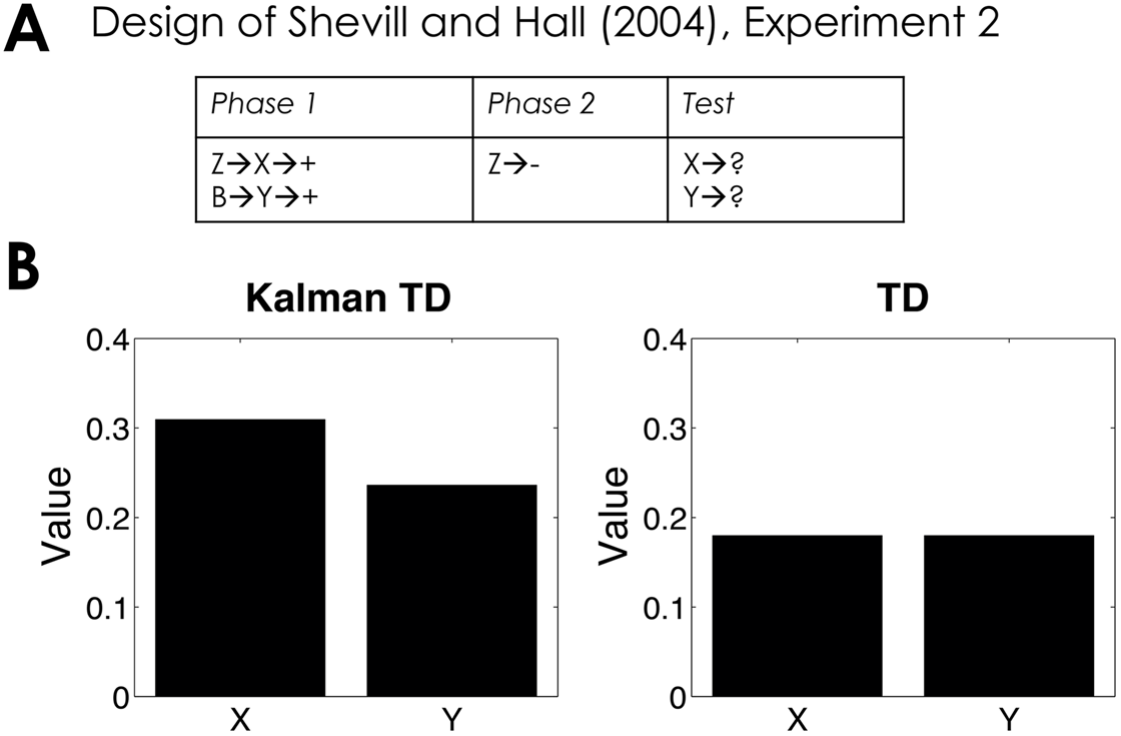
Recovery from overshadowing. (*A*) Experimental design [62]. (*B*) Simulated value of stimulus X and stimulus Y computed by Kalman TD (left) and TD (right).

A conceptually related design was studied by Shevill and Hall [62]. Instead of extinguishing the first-order stimulus X, they extinguished the second-order stimulus Z and examined the effect on responding to the first-order stimulus (Fig 8A). This extinction procedure increased responding to the first-order stimulus relative to another first-order stimulus (Y) whose associated second-order stimulus had not been extinguished. This finding is predicted by Kalman TD, but not TD (Fig 8B), because in a serial conditioning procedure the first-order stimulus overshadows the second-order stimulus, and extinguishing the first-order stimulus causes a recovery from overshadowing (a reduced first-order value is evidence that the second-order stimulus was responsible for the outcome). Note that this explanation is essentially the same as the one provided by the Kalman filter for recovery from overshadowing with simultaneous compounds [34]; the key difference here is that in serial compounds the second-order stimulus tends to differentially overshadow the first-order stimulus [63].

## Discussion

While the theoretical literature on associative learning is vast and complex, a few principles continue to play a central role in contemporary thinking. Some of these principles are embodied in the Rescorla-Wagner model and its generalizations—the TD model and the Bayesian Kalman filter model. Each model has strengths and weaknesses, as reviewed above. I have argued that Kalman TD represents a synthesis of these models that combines their strengths and remedies some of their weaknesses.

These models are by no means the only generalizations of the Rescorla-Wagner model (see, for example, [18, 64]), and there are other theoretical frameworks that offer different perspectives on the mechanisms underlying associative learning (e.g., [5, 7, 8, 43, 65]). Nonetheless, the synthesis of Bayesian and TD models has special significance given their influence on contemporary experimental research, particularly in neuroscience [48, 66]. These models offer different normative views of the associative learning problem—the Kalman filter views associative learning as tracking a changing reward distribution over time, while the TD model views associative learning as predicting long-term future reward (value). A central goal of this paper was to provide a unifying view, according to which associative learning is the tracking of a changing value distribution over time. The fruit of this unification is a model that can account for a number of complex phenomena that cannot be accounted for by either model on its own.

While Kalman TD can capture a number of phenomena qualitatively, a task for future research is to validate the model’s quantitative predictions. Such a validation is hampered by the fact that associative learning paradigms differ in many procedural details. Thus, it is important to adopt a single paradigm whose parameters can be explored systematically. Quantitative evaluation of Kalman filtering has been extensively studied in the motor control literature [67], and similar experimental techniques could be applied to associative learning. Among the predictions made by Kalman TD are: (1) uncertainty should grow linearly with the intertrial interval, and (2) the strength of association should grow linearly with the magnitude of the temporal derivative of the features.

### Limitations and extensions

One of the important insights of the Pearce-Hall model [17] was that learning rate should increase with surprise—formalized as the absolute value of recent prediction errors. This model successfully predicts that inconsistently pairing a CS with an outcome enhances its learning rate in a subsequent training phase with a different outcome [68]. In the Kalman filter (as well as in Kalman TD), changes in learning rate are driven solely by changes in the covariance matrix, which does not depend on outcomes. Thus, the model cannot explain any changes in learning rate that depend on prediction errors.

One way to deal with this problem is to recognize that the animal may have uncertainty about the transition dynamics (parameterized by *τ*), so that it learns simultaneously about the associative weights and *τ*. It is straightforward to show that the partial derivative of the log-likelihood with respect to *τ* monotonically increases with $\delta_{t}^{2}$, which means that gradient ascent will increase *τ* when the squared prediction error is greater than 0. This will give rise to qualitatively similar behavior to the Pearce-Hall model. Closely related Bayesian treatments have been recently explored, although not in the context of TD learning [38, 39, 69, 70].

Another issue that arises in models of associative learning is the problem of feature (or state space) representation [71]. When we present an animal with a stimulus configuration, it is reasonable to expect that the animal applies some kind of processing to the stimulus representation. Some neural network models conceive this processing as the application of a non-linear transformation to the stimulus inputs, resulting in a hidden-layer representation that encodes configural features [64, 72, 73]. Other models derive stimulus representation from a clustering process that partitions stimulus inputs into a discrete set of states [7, 71, 74, 75]. A related line of work has studied the representation of temporally extended stimuli; for example, a number of theories postulate a distributed representation of stimuli using basis functions with temporal receptive fields (see [52] for a review). In general, any of these representations are compatible with Kalman TD as long as values are linear functions of the representation. While this may sound limiting, it is in fact extremely powerful, since any smooth function can be arbitrarily well approximated by a linear combination of suitably chosen basis functions [76].

The final issue I will mention here concerns instrumental learning: A complete theory of associative learning must account for associations between actions and outcomes. One influential framework for combining Pavlovian and instrumental learning processes is the actor-critic architecture [77], according to which a Pavlovian “critic” learns state values, while an instrumental “actor” optimizes its policy using the critic’s prediction errors. Within this architecture, Kalman TD could function as a Bayesian critic. An interesting question that then arises is what role the critic’s uncertainty should play in guiding policy updating (see [78] for one possibility).

### Conclusions

This paper makes several contributions. First, it provides a unifying review of several associative learning models, elucidating their connections and their grounding in normative computational principles. Second, it presents new simulations that highlight previously unappreciated aspects of these models. Third, it presents Kalman TD, a synthesis of these models. While this model has been described in other papers [13, 14], this is the first systematic application to associative learning. This paper demonstrates that several prominent themes in associative learning theory can be coherently unified.

## Methods

### Simulation details

#### Latent learning

In the “Pre” condition, the agent was exposed to 10 pre-exposure trials (A→-) followed by 10 conditioning trials (A→+). In the “No-Pre” condition, the pre-exposure phase was omitted.

#### Overshadowing

In the “overshadowing” condition, the agent was exposed to 10 compound conditioning trials (AB→+) followed by a test of responding to B. In the “unovershadowing” condition, the agent was additionally exposed to 10 extinction trials (A→-) between conditioning and test.

#### Forward blocking

In the “blocking” condition, the agent was exposed to 10 conditioning trials (A→+) followed by 10 compound conditioning trials (AB→+) and a test of responding to B. In the “unblocking” condition, the agent was additionally exposed to 10 extinction trials (A→-) between compound conditioning and test.

#### Overexpectation

In the “overexpectation” condition, the agent was exposed to 10 conditioning trials for each stimulus (A→+ / B→+) followed by 10 compound conditioning trials (AB→+) and a test of responding to B. In the “unoverexpectation” condition, the agent was additionally exposed to 10 extinction trials (A→-) between compound conditioning and test.

#### Conditioned inhibition

In the “inhibition” condition, the agent was exposed to 10 A→+ trials and 10 AB→- trials, followed by a test of responding to B. In the “uninhibition” condition, the agent was additionally exposed to 10 extinction trials (A→-) prior to test.

#### Overshadowing and second-order conditioning

The design is summarized in Fig 4A. Each phase consisted of 10 trials.

#### Serial compound extinction and latent inhibition

The designs are summarized in Figs 6A and 7A. Each phase consisted of 10 trials.

#### Recovery from overshadowing

The design is summarized in Fig 8A. Each phase consisted of 10 trials.

### Model parameters

#### Kalman filter

For all simulations, the following parameters were used: $\sigma_{w}^{2}=1,\sigma_{r}^{2}=1,\tau^{2}=0.01$.

#### Temporal difference learning

For all simulations, the following parameters were used: *α* = 0.3, *γ* = 0.98. A *complete serial compound* [9, 48] was used for the temporal representation: Each stimulus was divided into 4 time bins, and each bin acted as a stimulus feature that was active only at a specific time relative to the stimulus onset. The precise duration of the stimuli was not important for our results.

#### Kalman temporal difference learning

For all simulations, the parameters were the same as for the Kalman filter, with the addition of a discount factor *γ* = 0.98. The temporal representation was the same complete serial compound used in the TD simulations.
