# Correction: A Unifying Probabilistic View of Associative Learning

**DOI:** 10.1371/journal.pcbi.1005829

**Published:** 2017-11-16

**Authors:** The PLOS Computational Biology Staff

There is an error in equation 15, the “discounted time derivative” h_t_ is defined incorrectly. It should read as follows:

“Operationally, the only change from the Kalman filter model described above is to replace the stimulus features x_t_ with their discounted time derivative, h_t_ = x_t_ - γx_t+1_. To see why this makes sense, note that the immediate reward can be expressed in terms of the difference between two values:

$$\begin{array}{l} r_{t}=V\left(x_{t}\right)-\gamma V\left(x_{t+1}\right)\\ =w_{t}x_{t}-\gamma w_{t}x_{t+1}\\ =w_{t}\left(x_{t}-\gamma x_{t+1}\right). \end{array}$$ (1)

This error does not affect the simulations, which were implemented with the correct definition.
